# Fever as a Cause of Hypophosphatemia in Patients with Malaria

**DOI:** 10.1371/journal.pone.0001380

**Published:** 2007-12-26

**Authors:** Richard Haber, Warren Browner

**Affiliations:** 1 Department of Medicine, University of California, San Francisco, United States of America; 2 California Pacific Medical Center, San Francisco, California, United States of America; Mahidol University, Thailand

## Abstract

Hypophosphatemia occurs in 40 to 60% of patients with acute malaria, and in many other conditions associated with elevations of body temperature. To determine the prevalence and causes of hypophosphatemia in patients with malaria, we retrospectively studied all adults diagnosed with acute malaria during a 12-year period. To validate our findings, we analyzed a second sample of malaria patients during a subsequent 10-year period. Serum phosphorus correlated inversely with temperature (n = 59, r = −0.62; P<0.0001), such that each 1°C increase in body temperature was associated with a reduction of 0.18 mmol/L (0.56 mg/dL) in the serum phosphorus level (95% confidence interval: −0.12 to −0.24 mmol/L [−0.37 to −0.74 mg/dL] per 1°C). A similar effect was observed among 19 patients who had repeat measurements of serum phosphorus and temperature. In a multiple linear regression analysis, the relation between temperature and serum phosphorus level was independent of blood pH, PCO2, and serum levels of potassium, bicarbonate, calcium, albumin, and glucose. Our study demonstrates a strong inverse linear relation between body temperature and serum phosphorus level that was not explained by other factors known to cause hypophosphatemia. If causal, this association can account for the high prevalence of hypophosphatemia, observed in our patients and in previous studies of patients with malaria. Because hypophosphatemia has been observed in other clinical conditions characterized by fever or hyperthermia, this relation may not be unique to malaria. Elevation of body temperature should be added to the list of causes of hypophosphatemia.

## Introduction

Low serum phosphorus levels (<0.81 mmol/L [2.5 mg/dL]) are common in hospitalized patients [1]. When combined with chronic phosphate depletion, hypophosphatemia can result in serious neurologic, cardiopulmonary, musculoskeletal, hematological, and metabolic dysfunction [1]–[3].

Although hypophosphatemia may occur in 40–60% of patients with acute malaria [4]–[6], its pathogenesis in this setting is not known. To determine the prevalence and causes of hypophosphatemia in patients with malaria, we retrospectively studied all adults diagnosed with acute malaria during a 12-year period. To validate our findings, we analyzed a second sample of malaria patients during a subsequent 10-year period.

## Methods

### Objectives

We sought to determine the correlation between body temperature and serum phosphorus level in patients with malaria, and to see whether that correlation was affected by adjustment for potential confounding variables, such as serum albumin or bicarbonate levels. We also determined whether changes in body temperature correlated with changes in serum phosphorus levels.

### Participants

Medical records of all patients 18 years of age or greater presenting to San Francisco General Hospital with acute malaria between 1976 and 1987 were reviewed. These dates were chosen because serum phosphorus (measured as phosphate, PO_4_) was included in the automated multichannel “metabolic” panel during that time. Patients were identified through medical records discharge diagnoses (ICD code 0.84.0-0.84.9) and the parasitology laboratory log. To validate the relation between body temperature and serum phosphorus observed in the first sample, data from a second group of patients who presented to San Francisco General Hospital and the University of California, San Francisco Moffitt-Long Hospitals during an additional 10-year period (1988–1997) were analyzed.

### Description of Procedures

We abstracted demographic and clinical information at presentation from the medical records, with particular attention to conditions associated with hypophosphatemia. Initial temperature and laboratory values (including serum levels of phosphorus, calcium, potassium, bicarbonate, glucose, albumin, creatinine, indirect bilirubin, aspartate aminotransferase and lactate dehydrogenase; a complete blood count with differential; and arterial blood gas values) were recorded; we verified that the samples had been obtained prior to initiation of any therapy. We also recorded subsequent “dyads” of body temperature and serum phosphorus levels if available; we required that the temperature have been recorded within four hours of the time that the serum sample was obtained.

### Ethics

The study was approved by the University of California, San Francisco Committee on Human Research; informed consent was not required. No external funds were used to support this study.

### Statistical methods

We compared patients with low and normal phosphorus levels using chi-squared and Student's *t* tests, as appropriate. Simple and multivariate linear regression models were used to determine the correlations between serum phosphorus levels (and changes in serum phosphorus levels) and other continuous variables, including arterial pH and PCO_2_ (since respiratory alkalosis can cause hypophosphatemia) [1], [2], [7]. Statistical significance was defined as P<0.05 (two-sided). All analyses were performed using StatCrunch software (www.statcrunch.com).

## Results

**Figure 1:**
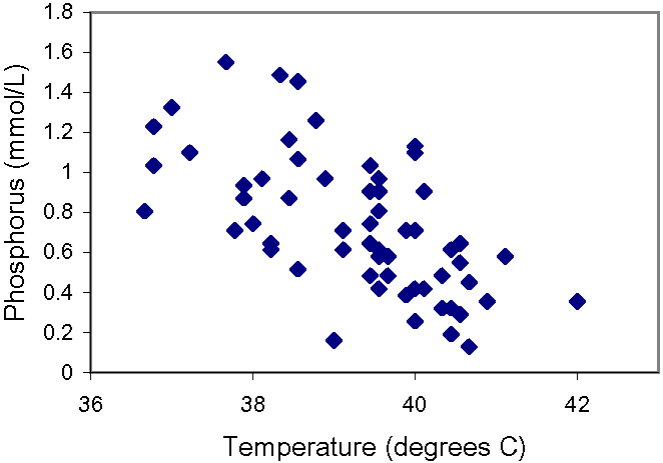
There was a highly significant correlation (r = −0.62, P<0.0001) between serum phosphorus level and temperature in 59 patients with malaria. Each 1°C increase in temperature was associated with a reduction in serum phosphorus level of 0.18 mmol/L [0.56 mg/dL].

Of the 76 adult patients who presented to San Francisco General Hospital with acute malaria in the initial 12-year period, 59 (78%) patients had serum phosphorus levels available for analysis, of whom 35 (59%) had low levels (<0.81 mmol/L [2.5 mg/dL]), including 5 (8%) with severe hypophosphatemia (<0.32 mmol/L [1.0 mg/dL ]). Patients with low serum phosphorus levels on admission had a significantly greater mean body temperature than those with normal levels (Table 1), and all 13 patients with temperatures ≥40.2°C had hypophosphatemia (Figure 1). There were also marginally significant differences in mean serum potassium and bicarbonate levels in those with and without hypophosphatemia (Table 1).

**Table 1:** Characteristics of the 59 subjects in the San Francisco General Hospital sample, stratified by serum phosphorus level.

Characteristic	Hypophosphatemia (<0.81 mmol/L [2.5 mg/dL]) (n = 37)	Normal phosphorus (n = 22)	P value
	N (%) or Mean±SD		
Female sex	10 (27)	9 (41)	0.41
Age (years)	31±15	29±10	0.44
Origin of malaria (Central America, India, SE Asia, Africa)	14 (38), 8 (22), 11 (30), 4 (11)	9 (41), 5 (23), 8 (36), 0 (0)	0.42
Plasmodium vivax	34 (92)	22 (100)	0.45
Temperature (°C)	39.7±1.0	38.5±1.0	<0.0001
Hemoglobin (g/L)	135±26	130±20	0.26
Glucose (mmol/L)	6.44±1.72	6.38±1.72	0.85
Potassium (mmol/L)	3.6±0.3	3.8±0.3	0.05
Bicarbonate (mmol/L)	22.8±2.4	23.9±2.9	0.05
Creatinine (umol/L)	97±18	88±18	0.26
Calcium (mmol/L)	2.2±0.1	2.2±0.2	0.96
Albumin (g/L)	39±4	39±4	0.93
Indirect bilirubin (mol/L)	24±12	21±10	0.46
Aspartate aminotransferase (U/L)	31±27	24±16	0.28
Lactate dehydrogenase (U/L)	239±65	257±97	0.41
pH*	7.44±0.04	7.46±0.03	0.28
PCO_2_ (mm Hg)*	32.6±2.8	34.2±3.5	0.19

* Arterial blood gas values were available for 17 patients, including 11 with hypophosphatemia.

Serum phosphorus correlated inversely with temperature (Figure 1, r = −0.62; P<0.0001), such that each 1°C increase in body temperature was associated with a reduction of 0.18 mmol/L [0.56 mg/dL] in the serum phosphorus level (95% confidence interval: −0.12 to −0.24 mmol/L [−0.37 to −0.74 mg/dL] per °C). In a multiple linear regression analysis, the relation between temperature and serum phosphorus was independent of serum levels of potassium, bicarbonate, calcium, albumin, and glucose (multivariate R^2^ = 0.48, P<0.0001 for temperature, all P≥0.32 for the other 5 variables).

**Figure 2:**
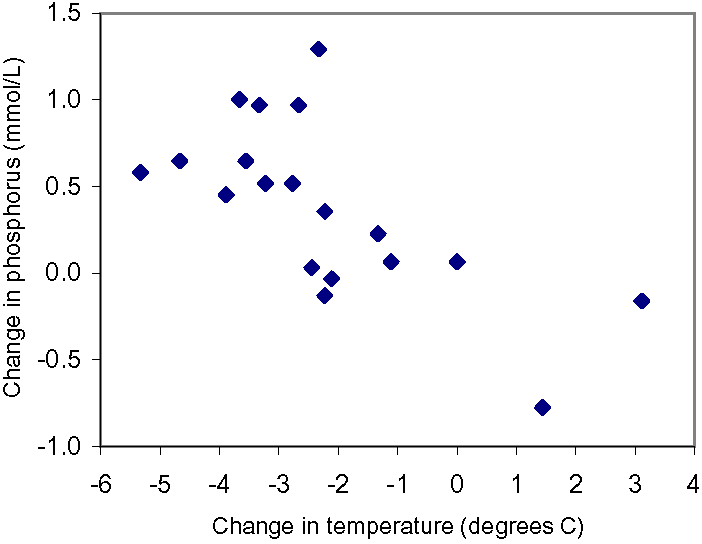
There was a highly significant correlation (r = −0.66, P<0.002) between changes in serum phosphorus level and changes in temperature in the 19 patients who had repeated measurements of both variables. For each 1°C decline in temperature, serum phosphorus level increased by 0.16 mmol/L [0.51 mg/dL].

Body temperature and serum phosphorus levels were available on two occasions in 19 patients. The correlation between the change in temperature and the change in serum phosphorus level was highly significant (Figure 2, r = −0.66, P<0.002). Each 1°C fall in body temperature was associated with a 0.16 mmol/L [0.51 mg/dL] (95% confidence interval: 0.07 to 0.26 mmol/L [0.23 to 0.79 mg/dL] per °C) increase in the serum phosphorus level. These changes occurred as rapidly as within four hours without specific phosphate replacement.

Arterial blood gas determinations were made in 17 patients (Table 1). There were no significant correlations between serum phosphorus levels and pH, PCO_2_, or bicarbonate levels (all P≥0.11). The inverse correlation between body temperature and serum phosphorus remained significant in this smaller group of patients even after adjusting for those measurements (r^2^ = 0.49, P<0.02).

### Validation sample

We analyzed a second sample of 121 patients presenting to San Francisco General Hospital and UCSF Moffitt-Long Hospitals during an additional 10-year period. Because serum phosphorus had been removed from the laboratory's automated multichannel panel during this time, there were many fewer patients in whom serum phosphorus levels were available (10 dyads from 6 patients). Nonetheless, even in this much smaller sample, the significant inverse linear relation between body temperature and serum phosphorus persisted, with a remarkably similar correlation (r = −0.67, P<0.0001).

### Possible adverse effects of hypophosphatemia

Because hypophosphatemia can cause hemolysis and rhabdomyolysis [1]–[3], we compared hematocrit and levels of hemoglobin, lactic dehydrogenase, indirect bilirubin, and aminotransferase in those with and without low serum phosphorus (Table 1). We found no significant difference in these values between the low and normal phosphorus groups and no correlation between these laboratory parameters and serum phosphorus level (P≥0.15).

### Results of chart reviews

Hypophosphatemia was not explained by other factors known to influence serum phosphorus, including carbohydrate, antacid or electrolyte administration, alcoholism or diabetes. There was no evidence for malabsorption, poor oral intake, conditions associated with enhanced renal excretion of phosphorus or conditions associated with intracellular phosphorus shifts such as respiratory alkalosis or carbohydrate administration.

## Discussion

Elevated body temperature is not listed as a cause of hypophosphatemia in reviews of this topic [1]–[3], nor has a linear relation between body temperature and serum phosphorus been reported previously. However, hypophosphatemia has been observed consistently in patients with conditions characterized by fever (altered hypothalamic set point) or hyperthermia (inability to dissipate a heat load), suggesting that the correlation between body temperature and serum phosphorus may not be unique to malaria. Whether the hypophosphatemia observed in these conditions is linearly related to body temperature has not been studied.

Indeed, hypophosphatemia has been observed in every experimental or pathologic condition associated with an increase in body temperature in which serum phosphorus has been reported; in both animals [8]–[13] and in humans [4]–[6], [14]–[29]. Hypophosphatemia has been reported in patients with fever from infection [bacteremia and sepsis [14]–[15], bacterial pneumonia [16], Legionnaire's disease [17], toxic shock syndrome [18], and malaria [4]–[6]]; in patients with fever from non-infectious causes [trauma, post operative, burns, and pancreatitis [19], drug fever [20]]; in volunteers undergoing experimental hyperthermia [21]–[22]; in patients receiving therapeutic hyperthermia [23]–[25]; in patients with accidental hyperthermia (heat stroke) [26]; during recovery from hypothermia [27]; and in patients with neuroleptic malignant syndrome [28] and Reye's syndrome [29]. If the association between body temperature and serum phosphorus levels is causal, and not restricted to patients with malaria, then this would provide a unifying explanation for the hypophosphatemia seen in these otherwise unrelated conditions.

There are three major causes of hypophosphatemia [1], [2]: redistribution of phosphorus from the extracellular fluid into cells, decreased intestinal absorption of phosphorus, and increased urinary phosphorus excretion. Our patients were young and previously healthy. None had renal or gastrointestinal disease, alcoholism, or other conditions, such as respiratory alkalosis or carbohydrate administration, that are known to cause phosphorus redistribution. The resolution of the hypophosphatemia without specific therapy was rapid as fever resolved, suggesting that redistribution of phosphorus between the extracellular and intracellular compartments may have accounted for the development and quick resolution of the hypophosphatemia.

Acute respiratory alkalosis, by increasing intracellular pH, accelerating glycolysis, and causing phosphorus to shift from the extracellular to the intracellular compartment [30], can result in transient hypophosphatemia in normal volunteers [7]. Because hyperventilation and acute respiratory alkalosis have been observed in sepsis [31] and in experimental [21], [22], [32], therapeutic [23], [24] and accidental hyperthermia (heat stroke) [25], this mechanism has been proposed as an explanation for the hypophosphatemia observed in these conditions [1]–[3]. In our patients, however, pH and pCO2 were only minimally altered even in those with low serum phosphorus levels, and there were no correlations between serum phosphorus and respiratory rate, pH, or pCO2. In addition, hypophosphatemia can occur in patients with bacterial pneumonia without respiratory alkalosis [16], and in patients undergoing therapeutic hyperthermia even when respiratory alkalosis is prevented by control of ventilation [24], suggesting that respiratory alkalosis is unlikely to be the sole explanation for the observed hypophosphatemia.

Carbohydrate administration, which can cause hypophosphatemia by increasing insulin secretion, accelerating glycolysis, and inducing intracellular shifts of phosphate [1]–[3], [33], is a common cause of hypophosphatemia in hospitalized patients [34], [35]. In our study, however, glucose levels did not differ between those with and without hypophosphatemia (see Table 1), and there was no significant correlation between serum phosphorus and glucose levels. In addition, blood samples were obtained before intravenous fluids were administered.

There is one report of a transient increase in the renal fractional excretion of phosphorus in malaria that correlated with a decrease in serum phosphorus levels; this was proposed as the mechanism by which malaria causes hypophosphatemia [5]. However, it is not clear that the degree of observed changes in fractional excretion of phosphorus can account for the magnitude and rapidity of observed changes in serum phosphorus levels. In addition, patients in that study were receiving intravenous therapy with glucose-containing fluids when serum and urine phosphorus measurements were obtained. Since carbohydrate administration can cause transient hypophosphatemia [1]–[3], [33] and volume expansion can increase renal excretion of phosphorus [1], [2], [36], it is unclear whether the increased renal phosphorus excretion was responsible for the observed hypophosphatemia.

Measurements of urinary phosphorus excretion in patients undergoing therapeutic hyperthermia—in which transient and severe hypophosphatemia is common—have yielded conflicting results. One study reported increased renal excretion of phosphorus [24], while another reported decreased excretion [23]; the differences may depend on whether respiratory alkalosis was induced (decreased excretion) or prevented (increased excretion). These data suggest that increased renal excretion of phosphorus is not the primary cause for the hypophosphatemia observed in these patients, since hypophosphatemia occurred even when renal phosphorus excretion was decreased. Because we did not measure urinary phosphorus, we cannot assess the effects of changes in renal phosphorus excretion in our patients.

We hypothesize that elevation of body temperature increases intracellular utilization of phosphate in the glycolytic pathway, causing phosphorus to shift from the extracellular fluid into cells. For example, in one study of human volunteers, a 2°C rise in temperature increased oxygen consumption by 19% [37]; in another study, the same rise in temperature was accompanied by a 26% increase in oxygen consumption and a 23% increase in metabolic rate [38]. In rat cerebral cortex, a 1°C rise in temperature results in a 5–6% increase in oxygen consumption, an increase in the tissue to serum glucose ratio, and an increase in intracellular glucose 6 phosphate [39]. Similarly, rhesus monkeys infected with *Plasmodium coatneyi* showed both hypophosphatemia and an increased arteriovenous difference in glucose concentration, consistent with increased glucose utilization [8]. A positive correlation between glucose disposal and oral temperature (r = 0.65, P<0.03) has also been observed in humans with malaria [40]. Because increases in cellular metabolism are linearly related to temperature, the higher the temperature, the greater the expected intracellular shift in phosphorus.

The correlation between body temperature and serum phosphorus might be caused by non-thermogenic effects of pro-inflammatory cytokines, such as interleukin (IL)-1, IL-6, tumor necrosis factor (TNF)-α, and interferon-γ. Increased levels of these cytokines occur in patients with malaria [6], [41]–[44], other infectious and non-infectious causes of fever [15], [45], [46] and hyperthermia [26]. In one study of patients with sepsis [15], cytokine levels were inversely related to serum phosphorus, although their relations with body temperature were not reported. In mice, injection of IL-1B, IL-6, or TNF-α leads to a significant fall in serum phosphorus levels [15]. Similarly, severe hypophosphatemia was induced by infusion of recombinant TNF in 22 patients with liver metastases [47]. Whether this is a direct effect, or one that is mediated through a cytokine-induced increase in body temperature, is not known. This question might be resolved by studying genetic models (e.g., cyclooxygenase [COX]-2 knockout mice) in which injection of IL-6 does not cause fever [48]. Indeed, in experimental acute bovine mastitis, both fever and hypophosphatemia can be prevented by treating animals with ibuprofen [9].

In our patients, there were no differences in serum levels of indirect bilirubin, lactic dehydrogenase, hematocrit, hemoglobin, creatinine, or aminotransferase among patients with and without hypophosphatemia, suggesting that the hypophosphatemia did not result in measurable hemolysis or rhabdomyolysis. This is consistent with other data indicating that intracellular shifts of phosphate do not cause cellular damage in the absence of chronic phosphate depletion [1]–[3].

### Limitations

Our study has several limitations. First, our findings, although validated in two separate samples, are restricted to patients with malaria. Whether these findings are applicable to patients with elevated body temperature from other causes remains to be demonstrated. Second, since nearly all of our patients had *P. vivax* malaria, in whom severe parasitemia is rare (and parasite loads were not available to us). Thus we could not determine whether the degree of parasitemia affected temperature or phosphate levels. Third, although we postulate that the major mechanism for hypophosphatemia in our patients was a temperature-induced intracellular shift of phosphorus, we did not measure renal excretion of phosphorus, so we cannot exclude the possibility of renal loss. Fourth, we did not measure hormones that can affect serum phosphorus such as parathyroid hormone, Vitamin D, and the phosphatonins (e.g., fibroblast growth factor 23) [1]. Thus we cannot comment on the effects of hormonal changes, if any, on the observed hypophosphatemia. Finally, we used available clinical data; for example, we did not have information on whether temperature was measured orally or rectally, and the instruments used to measure serum phosphate levels likely changed during the several-year periods of study. However, we did find a strong correlation between temperature and phosphate levels; had the data been measured more precisely and accurately, the correlation would almost certainly have been even stronger and more significant.

In sum, our study demonstrates a strong inverse linear relation between body temperature and serum phosphorus level that was not explained by other factors known to cause hypophosphatemia. If causal, this association can account for the high prevalence of hypophosphatemia and the rapid changes in serum phosphorus levels observed in our patients and in previous studies of patients with malaria. Because hypophosphatemia has been observed in other clinical conditions characterized by fever or hyperthermia, this relation may not be unique to malaria or to the mechanism of body temperature elevation. Indeed, a direct effect of elevated temperature may provide a unifying hypothesis to explain the hypophosphatemia observed in other conditions associated with hyperthermia or fever. Regardless of its pathogenesis, because each 1°C rise in temperature was associated with about a 0.18 mmol/L [0.56 mg/dL] reduction in serum phosphorus levels, knowledge of body temperature is important in evaluating patients with low serum phosphorus levels. Elevation of body temperature should be added to the list of possible causes of hypophosphatemia.
